# Plasma membrane calcium ATPase (PMCA4): A housekeeper for RT-PCR relative quantification of polytopic membrane proteins

**DOI:** 10.1186/1471-2199-7-29

**Published:** 2006-09-17

**Authors:** Anna Maria Calcagno, Katherine J Chewning, Chung-Pu Wu, Suresh V Ambudkar

**Affiliations:** 1 Laboratory of Cell Biology, Center for Cancer Research, National Cancer Institute, NIH, DHHS, Bethesda, MD 20892–42546, USA

## Abstract

**Background:**

Although relative quantification of real-time RT-PCR data can provide valuable information, one limitation remains the selection of an appropriate reference gene. No one gene has emerged as a universal reference gene and much debate surrounds some of the more commonly used reference genes, such as glyceraldehyde-3-phosphate dehydrogenase (GAPDH). At this time, no gene encoding for a plasma membrane protein serves as a reference gene, and relative quantification of plasma membrane proteins is performed with genes encoding soluble proteins, which differ greatly in quantity and in targeting and trafficking from plasma membrane proteins. In this work, our aim was to identify a housekeeping gene, ideally one that codes for a plasma membrane protein, whose expression remains the same regardless of drug treatment and across a wide range of tissues to be used for relative quantification of real-time RT-PCR data for ATP binding cassette (ABC) plasma membrane transporters.

**Results:**

In studies evaluating the expression levels of two commonly used reference genes coding for soluble proteins and two genes coding for membrane proteins, one plasma membrane protein, plasma membrane calcium-ATPase 4 (PMCA4), was comparable to the two reference genes already in use. In addition, PMCA4 expression shows little variation across eight drug-treated cell lines and was found to be superior to GAPDH and HPRT1, commonly used reference genes. Finally, we show PMCA4 used as a reference gene for normalizing ABC transporter expression in a drug-resistant lung carcinoma cell line.

**Conclusion:**

We have found that PMCA4 is a good housekeeping gene for normalization of gene expression for polytopic membrane proteins including transporters and receptors.

## Background

Relative quantification for real-time RT-PCR requires the use of a reference gene for normalization [1]. An "ideal" reference gene should be expressed at similar levels in different cell types and under various treatment conditions. Many commonly used reference genes or "housekeeping" genes do not always possess these two necessary attributes. Several commonly used housekeeping genes were originally used as references for more qualitative assays such as Northern blots and conventional RT-PCR, and their use in quantitative RT-PCR was not originally re-evaluated[2]. Therefore, the selection of the appropriate reference gene for performing relative quantification has been a topic of much discussion and evaluation [1,3,4]. This becomes more critical when examining the mRNA expression of membrane proteins, since many of the frequently used reference genes are soluble proteins, which differ significantly in total amount and in targeting and trafficking from membrane proteins.

Membrane proteins are a major component of the human genome, as they comprise nearly 30% of the total human genome [5]. These proteins are also involved in approximately 85% of cell signalling pathways. The biogenesis of membrane proteins and cytosolic proteins varies greatly. At transcription, specific mRNA-binding proteins are added to nascent mRNA to ensure RNA integrity and to oversee RNA export from the nucleus, subcellular localization, translation and stability [6]. Membrane localization of mRNA has been reported in membrane proteins, and this localization may facilitate cotranslational import of some membrane transporters as is seen in Atm1, a yeast ABC transporter of the inner mitochondrial membrane, and Ist2, a yeast plasma membrane putative ion channel [7]. Investigators have also shown that mRNAs that encode for secreted or membrane proteins are preferentially found in membrane-bound polysomes [8]. The translation of secretory, integral membrane proteins as well as that of luminal and membrane proteins of the endoplasmic reticulum (ER) take place on membrane-bound ribosomes of the ER [9], whereas the synthesis of cytosolic proteins occurs on the ribosomes within the cytoplasm. Polytopic membrane proteins have multiple membrane spanning domains, and they are believed to cotranslationally incorporate into the membrane of the ER with the assistance of the translocon [10].

An important superfamily of membrane proteins is the ATP-binding cassette (ABC) transporter family. Several ABC transporters are linked to disease conditions in humans including cystic fibrosis, Stargardt disease, Dubin-Johnson syndrome, Pseudoxanthoma elasticum and adrenoleukodystrophy [11]. In addition, the overexpression of ABC transporters is the predominant cause of multidrug resistance in cancer [12], and understanding the triggers for overexpression of these proteins upon exposure to various drug regimens would help researchers find ways to circumvent multidrug resistance. Therefore, a reliable reference gene that does not change following drug treatment is vital for the relative quantification of ABC transporter gene expression. In this work, our goal was to identify a housekeeping gene, ideally one that codes for a plasma membrane protein, whose expression remains the same regardless of drug treatment and across a wide range of tissues for normalization of real-time RT-PCR data for ABC transporters.

We hypothesized that plasma membrane proteins could function as reference genes and that a plasma membrane reference gene would be most appropriate for relative quantification of membrane proteins consisting of 4–12 membrane spanning helices otherwise known as polytopic membrane proteins. As mentioned previously, membrane protein biogenesis, in particular for polytopic membrane proteins, involves several steps that are specific to membrane proteins; therefore, a reference gene encoding for a protein which undergoes similar process of biogenesis would be best to use as a reference measurement for gene expression. First of all, to evaluate this hypothesis, two commonly used soluble protein reference genes, glyceraldehyde-3-phosphate dehydrogenase (GAPDH) and hypoxanthine phosphoribosyl transferase 1 (HPRT1), and two commonly expressed plasma membrane proteins, plasma membrane calcium-ATPase 4 (PMCA4) and the alpha subunit of the Na^+ ^/K^+^-ATPase (ATP1A1), were evaluated using real-time RT-PCR with a variety of human tissue RNA samples. PMCA4 and ATP1A1 were chosen because both are ubiquitously expressed polytopic plasma membrane proteins. We determined that the consistency of PMCA4's expression profile was comparable to that of the commonly used reference genes across a panel of tissues. We then compared these four genes in eight drug-treated cell lines, and PMCA4 was found to be superior to GAPDH and HPRT1. Finally, we used PMCA4 to normalize ABC transporter expression from real-time RT-PCR for drug-resistant lung carcinoma cells.

## Results and Discussion

### PMCA4 shows comparable expression to GAPDH and HPRT1 in various tissues

**Figure 1:**
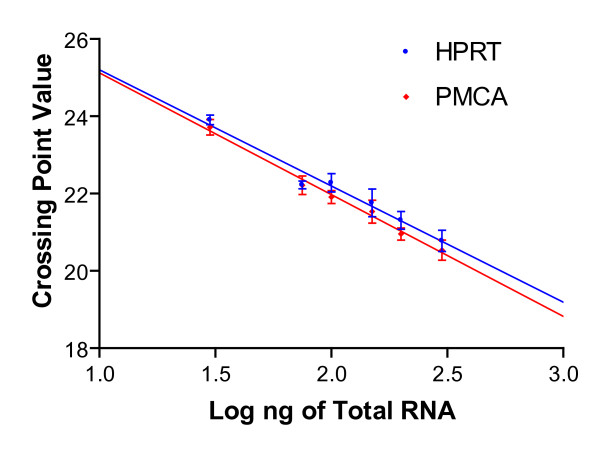
**Standard curve to evaluate efficiency of RT-PCR reaction**. The HPRT1 (blue circle) and PMCA4 (red diamond) primers were evaluated over a range of total RNA concentrations (30 ng-300 ng total RNA). The average crossing point values are given for the n = 4, and the error bars represent ± SD.

To determine the optimal concentration of total RNA to use within our studies, the efficiency of the RT-PCR reaction was evaluated over a concentration range. The standard curve for two primers for the detection of HPRT1 and PMCA4 indicates that the efficiency is nearly 2 (Fig. 1). Two hundred nanograms of total RNA was found to be within the linear range of the reaction, and this quantity was used to evaluate the primers for this and all subsequent studies comparing the four genes.

**Figure 2:**
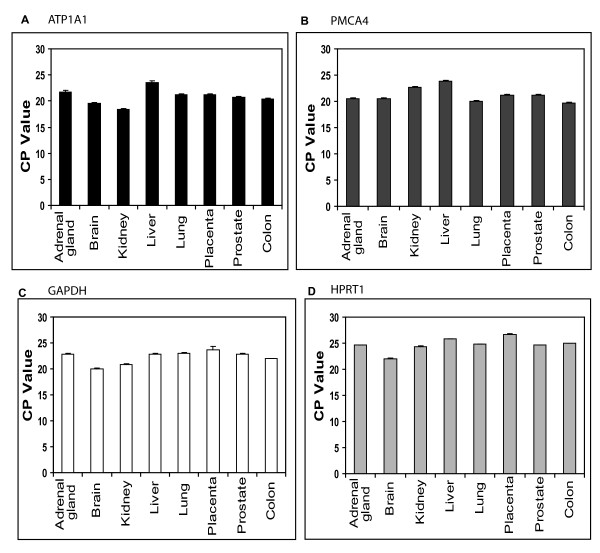
**Quantification of commonly expressed genes encoding soluble or membrane proteins in various human tissue samples using real time RT-PCR**. The expression levels of two polytopic membrane protein genes (panel A and B) and two soluble protein genes (panel C and D) in eight human tissue samples known to express ABC transporters were evaluated using RT-PCR. A) ATP1A1; B) PMCA4; C) GAPDH; and D) HPRT1. The error bars represent standard deviation (n = 4).

To evaluate the use of ATP1A1 or PMCA4 as reference genes, their expression patterns for a variety of human tissue RNA samples were compared to those of two commonly used reference genes, GAPDH and HPRT1. The average crossing point values determined by real-time RT-PCR for the 4 housekeeping genes across different tissue samples with standard deviation are shown in Fig. 2. The selected tissue types are known to express ABC transporters at moderate to high levels. GAPDH and HPRT1 showed the least amount of variation for the panel of tissues tested (Mean = 22.24 and variance = 1.26; Mean = 24.75 and variance = 1.64, respectively). However, similar to GAPDH and HPRT1, PMCA4, which encodes a plasma membrane calcium pump that removes bivalent calcium ions from cells against very large concentration gradients [13-16], is consistently expressed in this varying panel of tissues (Mean = 21.17 and variance = 1.87). ATP1A1, the alpha subunit of the plasma membrane Na^+ ^/K^+ ^ATPase [17], shows a greater variance in expression compared to either PMCA4 or the commonly used reference genes (Mean = 20.84 and variance = 2.09). This suggests that unlike PMCA4, ATP1A1 is not the best choice for tissue samples as a reference gene. Further statistical analysis using a one-sided Fisher variance-ratio, comparing the variance for the PMCA4 samples to the variance for each other gene samples separately, indicates that there is no statistically significant difference (p > 0.05) between PMCA4 expression and that of the other genes (ATP1A1 p = 0.383; GAPDH p = 0.187; and HPRT1 p = 0.356). The variances for the ATP1A1 samples were also compared to the variances for the GAPDH and HPRT1 samples; the p values were 0.118 and 0.252, respectively. Additional studies with cell culture lines from other tissue types generated similar results (Table 1). The F-test for the cell culture lines did indicate that the variance of the ATP1A1 expression profile is statistically different than that of PMCA4 (p = 0.041), whereas there is no statistically significant difference between the variances of the PMCA4 and the GAPDH samples (p = 0.093) or the variances of the PMCA4 and the HPRT1 samples (p = 0.074). There was also a statistically significant difference between the variance of ATP1A1 and those of GAPDH and HPRT1 (both p values = 0.001). This suggests that PMCA4 is comparable to two established reference genes, GAPDH and HPRT1, while ATP1A1 is not.

### PMCA4 expression is unchanged under a variety of treatment conditions

**Figure 3:**
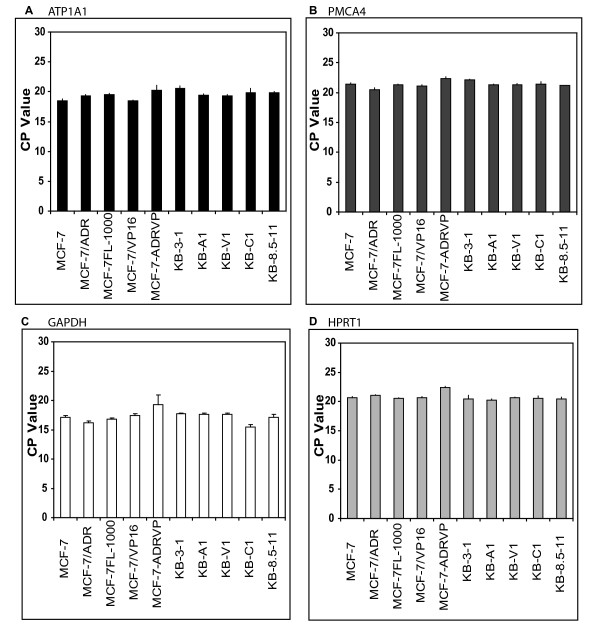
**Comparison of reference gene expression in drug-resistant cell lines**. The two parental cell lines, MCF-7 and KB-3-1, and several of their drug-resistant subclones were evaluated using RT-PCR. The drug-resistant MCF-7 cell lines were as follows: MCF-7/ADR (doxorubicin-treated)[18]; MCF-7FL-1000 (flavopiridol-treated)[20]; MCF-7/VP16 (etoposide-treated) [19]; and MCF-7-ADRVP (adriamycin- and verapamil-treated)[21]. The KB cell lines were KB-A1 (doxorubicin-treated); KB-V1 (vinblastine-selected) and KB-C1 and KB-8.5-11 (colchicine-selected)[22]. A) ATP1A1; B) PMCA4; C) GAPDH; and D) HPRT1. The error bars represent standard deviation (n = 4).

Reference genes must be expressed at similar levels in different cell types and more importantly must remain consistent under various treatment conditions. Our previous results address tissue expression of PMCA4. In an attempt to further examine the utility of PMCA4 as a reference gene; its expression was evaluated in 8 drug-treated cell lines. RNA was isolated from four drug-treated sublines and from the drug-sensitive parental cells, MCF-7 and KB-3-1. The MCF-7 (human breast adenocarcinoma) sublines were the following: MCF-7/ADR [18], MCF-7/VP16 [19], MCF-7FL-1000 [20], and MCF-7-ADRVP [21]. The KB-3-1 (human cervical squamous cell carcinoma) sublines were selected with either doxorubicin (KB-A1), vinblastine (KB-V1) or colchicine (KB-C1 and KB-8.5-11) [22]. These drug-selected cell lines are known to overexpress ABC transporters at the mRNA level. We also re-evaluated ABC transporter expression levels within these lines using RT-PCR to confirm that overexpression was present (data not shown). The average crossing point values are given for the different cell lines in Fig. 3. Interestingly, in this analysis, GAPDH, a commonly used reference gene, performed poorly under these conditions (Mean = 17.24 and variance = 1.21), and PMCA4 expression was the most consistent across the various high dose drug treatments (Mean = 21.4 and variance = 0.29). HPRT1 showed slightly more variation than PMCA4 (Mean = 20.77 and variance = 0.41). Again, the results for ATP1A1 (Mean = 19.49 and variance = 0.55) show that the variance is nearly two-fold larger than that for PMCA4. This taken together with the results from the tissue and cell line expression studies suggests that ATP1A1 is not suitable as a reference gene.

PMCA4, on the other hand, was found to be superior to all other reference genes tested in this study. An F-test was used to individually compare the variance of the PMCA4 samples to the variance of the samples for the other genes. The variances for ATP1A1 and GAPDH, p = 0.027 and 0.00001, respectively, were statistically different than the variance for PMCA4. No statistical difference was found between PMCA4 and HPRT1, p = 0.146. From these results, we first identified a reference gene which was foremost, a membrane protein and which would also be consistent for all cell lines examined, regardless of drug selection conditions. PMCA4 was chosen as the reference gene for our subsequent work because of the convincing data from these studies. To our knowledge, this is the first report of the use of a gene encoding a membrane protein for normalization of real time RT-PCR data for polytopic membrane transport proteins.

### PMCA4 is a good reference gene for polytopic plasma membrane proteins

**Figure 4:**
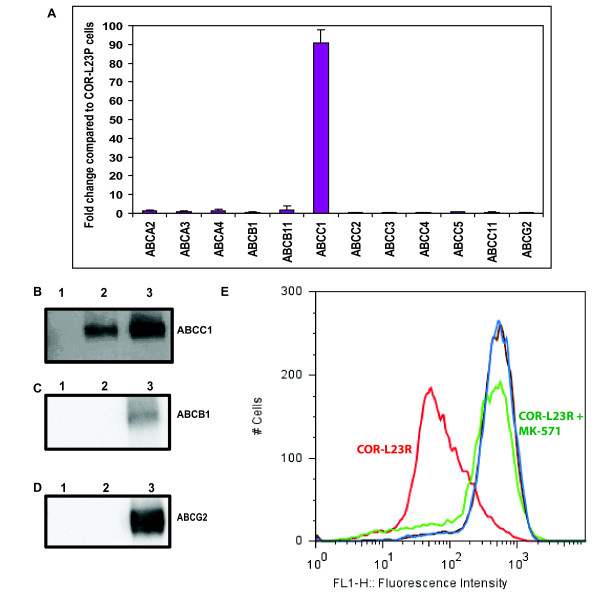
**Evaluation of selected ABC transporter expression in lung carcinoma COR-L23 (parental and resistant) cell lines**. A) The fold change in expression in COR-L23R cells compared to the parental cell line is depicted for the gene expression of ABC transporters. Two individual determinations of crossing point values are given. B) Western blotting with MRPr1 antibody (1:1000) shows that only COR-L23R cell lysates express ABCC1[27]. Lane 1, 20 μg of COR-L23P; lane 2, 10 μg of COR-L23R; and lane 3, 20 μg of COR-L23R. C) C219 (1:1000) was used to detect ABCB1[28]. Lane 1, COR-L23P (100,000 cells); lane 2, COR-L23R (100,000 cells); and lane 3, positive control, KB-A1 (100,000 cells). D) ABCG2 was detected with BXP-21 (1:1000)[29]. Lane 1, COR-L23P (100,000 cells); lane 2, COR-L23R (100,000 cells); and lane 3, positive control, MCF-7-FL1000 (100,000 cells). E) Functional ABCC1 is expressed in COR-L23R cells as determined by calcein-AM efflux assays. Brown solid line histogram, COR-L23P; blue solid line histogram, COR-L23P with 20 μM MK-571; red solid line histogram, COR-L23R control; and green solid line histogram, COR-L23R with 20 μM MK-571. The results from a typical experiment are shown in the panel. Similar results were obtained on two additional experiments.

We have shown that PMCA4 expression is consistent and that it should be used as a reference or housekeeping gene for membrane proteins. To demonstrate the use of PMCA4 for normalizing the ABC transporter expression profile for multidrug resistant cells, we evaluated parental COR-L23P and drug-resistant COR-L23R cells. COR-L23R was the only doxorubicin-resistant cell line reported to overexpress the ABC transporter, ABCC1, by step-wise selection using high doses of doxorubicin. Further characterization of multidrug resistance-linked ABC transporter gene expression has not been reported to date for the COR-L23R cells. Crossing point values for each transporter were normalized to the respective crossing point values for PMCA4. Data are presented as a fold change in gene expression for the drug-resistant cell line compared to the parental cells using the delta delta Ct method. Surprisingly, our results show that only this ABC transporter is highly overexpressed in the resistant cells. COR-L23R overexpresses ABCC1 (> 90-fold) with doxorubicin-selection compared to parental COR-L23P cells (Fig. 4), and no differences were found between COR-L23P and COR-L23R expression of PMCA4 (Cp = 21) at 300 ng of total RNA per reaction. However, if GAPDH was used as the reference gene, this fold change would be greatly inflated, since the crossing point values between COR-L23P (Cp = 18) and COR-L23R (Cp = 20) vary by 2 crossing point values. Alternatively, the mean expression value for HPRT1 for the COR-L23P cells was 19.8 while that of the COR-L23R cells was 20.1. Although these differences in Cp values appear to be minimal, the calculated fold change would be highly amplified when either GAPDH or HPRT1 was used with the delta-delta Ct method (Table 2).

No other ABC transporter in our panel (consisting of the 12 ABC transporters linked to multidrug resistance) was overexpressed, and only the transporter which was previously reported remained the ABC transporter conferring resistance as seen by Western blotting (Fig. 4C and 4D). ABCC1 was not detectable in the parental COR-L23P cells, as seen in this Western Blot. Calcein-AM efflux assays using MK-571, the typical ABCC1 inhibitor, further verified that COR-L23R cells overexpress functional ABCC1 (Fig. 4E). These results are consistent with previous reports on the drug resistance profiles of these cells [23].

In this work, we identified a consistent housekeeping gene, PMCA4, for plasma membrane proteins which was comparable to other established reference genes when examined in a diverse panel of tissues and cell lines. Our work investigated the effects of drug treatment and tissue type on the selection of a housekeeping gene; additional work evaluating other conditions is currently under way. PMCA4 was also found to be superior to these reference genes, since it showed less variation regardless of drug treatment for the KB-3-1 and MCF-7 cells. We also thoroughly characterized the ABC transporter gene expression profile for a multi-step doxorubicin-selected cell line using PMCA4 as the reference gene. Overexpression of ABCC1 was verified in the COR-L23R cells at both the mRNA and protein levels. Although we have found that PMCA4 works quite well for the described conditions, as for all known housekeeping genes, there are conditions where alternative housekeeping genes may work best. For PMCA4, other investigators reported that increased P_i _levels in cattle resulted in increased PMCA4 levels; neuronal development was also linked to increased PMCA4 levels [24,25]. PMCA4 appears to be a suitable housekeeping gene for normalization of gene expression for polytopic membrane proteins including transporters, ATPases and receptors.

## Methods

### Chemicals and other reagents

Acetoxy-methyl ester of calcein (calcein-AM) was purchased from Molecular Probes (Eugene, OR). Doxorubicin was purchased from Calbiochem (San Diego, CA). RT-PCR reagents were purchased from Roche Applied Sciences (Indianapolis, IN). RNA from various tissue types examined in Fig. 2 was purchased from Clontech (Mountain View, CA).

### Cell Lines

The human large-cell lung tumor line COR-L23P and its doxorubicin-selected MRP1-overexpressing multidrug-resistant variant COR-L23R were a generous gift from Margery A. Barrand (Department of Pharmacology, University of Cambridge, Cambridge, UK) [23]. Both COR-L23P and COR-L23R cells were cultured in RPMI 1640 medium with 10% (v/v) fetal calf serum supplemented with 100 units of penicillin/streptomycin/ml (Invitrogen) at 37°C in 5% CO_2 _humidified air. The COR-L23R cells were also maintained in the presence of 0.2 μg/ml doxorubicin.

The cervical epidermal carcinoma cell line KB-3-1 and its drug resistant subline, KB-A1, were generous gifts of Michael M. Gottesman (Laboratory of Cell Biology, National Cancer Institute, NIH, Bethesda, MD) [22], and the MCF-7 breast cancer cell line and the subline MCF-7/ADR were a generous gift of Kapil Mehta (MD Anderson Cancer Center, Houston, TX) [18]. These cell lines were cultured in DMEM with 10% (v/v) fetal calf serum supplemented with 2 mM glutamine and 100 units of penicillin/streptomycin/ml (Invitrogen) at 37°C in 5% CO_2 _humidified air. The drug resistant subline KB-A1 was kept under constant selection in 1 μg/ml doxorubicin while 0.5 μg/ml doxorubicin was added to the MCF-7/ADR cells every other passage.

### RNA Isolation

RNA was isolated from cells grown in 6-well plates to characterize reference gene expression in all cell lines and to characterize ABC transporter expression in the parental COR-L23P and drug-resistant COR-L23R cell lines. In these studies, 6-well plates were seeded at 200,000 cells per well for each cell type, and RNA was isolated following 72 hr of growth. Drug-resistant cell lines were plated with the appropriate concentration of drug during the 72 hr period. The medium and any detached cells were removed from the wells. RNA isolation was performed on the cells that remained attached using the Qiagen RNeasy kit (Valencia, CA) as per the manufacturer's protocol with a 15 minute on-column DNAse incubation step. RNA samples were isolated in triplicate. The pure RNA was quantified using the Nanodrop ND-1000 Spectrophotometer (Wilmington, DE). The integrity of the RNA was verified using the Agilent 2100 Bioanalyzer (Palo Alto, CA) with the Eukaryote Total RNA Nano assay. The RNA samples were stored at -80°C until needed.

### Quantitative RT-PCR

Real-time quantitative RT-PCR was used to measure the mRNA expression levels of the four reference gene candidates and the selected ABC transporters. The LightCycler RNA Master SYBR Green Kit and LightCycler II or LightCycler 480 instrument (Roche Biochemicals, Indianapolis, IN) were utilized in these studies. Primers for these genes were designed using the Roche Probe Design 2 software. All primer sets were tested prior to use in this work to ensure that only a single product of the correct size was amplified for all primer sets. The primer set for PMCA4 was designed to a region which is common to each of the splice variants and does not differentiate between them. (The list of primers is given in Table 3).

Two hundred nanograms of total RNA with 250 nM primers were used to evaluate the primers for the studies comparing the four reference genes. The LightCycler RNA Master SYBR Green Kit and LightCycler 480 instrument (Roche Biochemicals, Indianapolis, IN) were utilized in these studies to determine which of the four reference genes would be the most consistent across tissue types and treatment conditions. The average crossing point values for 4 housekeeping genes across different tissue samples with standard deviation were determined, and the RT-PCR conditions were as described in the manufacturer's protocol for the RNA Master SYBR Green kit. Negative controls consisting of no-template (water) reaction mixtures were run with all reactions.

The RT-PCR reaction for ABC transporters was performed on 300 ng total RNA with 250 nM specific primers under the following conditions in the LightCycler II: reverse transcription (20 min at 61°C), one cycle of denaturation at 95°C for 30 seconds, and PCR reaction of 45 cycles with denaturation (15s at 95°C), annealing (12s at 58°C), and elongation (30s at 72°C with a single fluorescence measurement). The PCR reaction was followed by a melting curve program (65–95°C with a heating rate of 0.1°C per second and a continuous fluorescence measurement) and then a cooling program at 40°C. Negative controls consisting of no-template (water) reaction mixtures were run with all reactions. PCR products were also run on agarose gels to confirm the formation of a single product at the desired size. Crossing points for each transcript were determined using the 2^nd ^derivative maximum analysis with the arithmetic baseline adjustment. Crossing point values for each transporter were normalized to the respective crossing point values for reference gene plasma membrane calcium ATPase 4. Data are presented as a fold change in gene expression for the drug resistant cell lines compared to the parental cells using the delta delta Ct method.

### Western Blotting

For the Western blotting assays, cells were harvested and the cell lysates were prepared from drug-resistant and parental cells using a lysis buffer containing 10 mM Tris, pH 8.0, 1% Triton X 100, 2 mM DTT, 1% aprotinin, 1 mM AEBSF, and DNase. Following brief sonication and the addition of SDS, equivalent numbers of cells were loaded and separated by 7% NuPAGE gels. The electrophoressed proteins from the gel were transferred to a nitrocellulose membrane and probed with the appropriate primary antibodies specific for the ABC protein of interest. Immunoblotting was performed using the C219 (1:2000) antibody for ABCB1, MRPr1 (1:1000) (Alexis) for ABCC1, Anti-BCRP (1:1000) for ABCG2, and the anti-mouse IgG-horseradish peroxidase (HRP) conjugated (1:10000) secondary antibody. HRP-dependent luminescence was developed using Western lighting chemiluminescence reagent plus (PerkinElmer, Wellesley, MA). The blot was exposed to Hyperfilm within a Hypercassette for various times. The intensity of bands on the blot was quantitated using a scanner and ImageQuant software (Piscataway, NJ).

### Assay of transport of fluorescent substrates by flow cytometry

A FACSort flow cytometer equipped with Cell Quest software (Becton-Dickinson, Franklin Lakes, NJ) was used for FACS analysis. Briefly, 300,000–500,000 cells were harvested after trypsinization by centrifugation at 500 × g and then resuspended in IMDM supplemented with 5% Fetal Bovine Serum. 0.25 μM calcein-AM was added to the cells in 4 ml of IMDM in the presence or absence of a specific inhibitor for the ABC transporter evaluated. For example, 20 μM MK571 was used to specifically inhibit ABCC1. The cells were incubated in a water bath at 37°C in the dark for 10 minutes for a calcein-AM efflux assay prior to being pelleted by centrifugation at 500 × g. The cell pellet was then resuspended in 300 μl of PBS containing 0.1 % BSA and then analyzed immediately using the flow cytometer as described previously [26].

### Statistics

Comparisons between the expression profiles for PMCA4 and each of the other genes was performed using a one-tailed F-test two sample for variances, with a confidence level of 0.05.

## Abbreviations

ABC, ATP binding cassette; calcein-AM, calcein acetoxy-methyl ester; MRP, multidrug resistance protein; MK-571, (3-(3-(2-(7-chloro-2-quinolinyl)ethenyl)phenyl) ((3-(dimethyl amino-3-oxo propyl)thio)methyl)thio)propanoic acid; PAGE, polyacrylamide gel electrophoresis; Pgp, P-glycoprotein; GAPDH, Glyceraldehyde-3-phosphate dehydrogenase; HPRT, Hypoxanthine phosphoribosyl transferase 1; PMCA4, Plasma Membrane Calcium- ATPase 4; ATP1A1, Na^+ ^/K^+^-ATPase

## Authors' contributions

AMC performed the RT-PCR studies and drafted the manuscript. KJC assisted AMC with these studies. CPW evaluated the COR-L23 cells by Western blotting and flow cytometry. SVA supervised the studies and finalized the manuscript. All authors read and approved the final manuscript.

## Figures and Tables

**Table 1:** Comparison of Housekeeping Gene Expression in Various Cell Lines^a^

**Cell line**	**Housekeeping Genes**			
	*ATP1A1 *^*b*^	*GAPDH*	*HPRT1*	*PMCA4*

Human Salivary Gland cells	19.62 ± 0.38	16.96 ± 0.11	21.33 ± 0.21	20.61 ± 0.14
HCT-116 (colorectal carcinoma)	20.4 ± 0.67	17.33 ± 0.4	21.71 ± 0.31	21.7 ± 0.36
KB-3-1 (Cervical squamous cell carcinoma)	20.57 ± 0.36	17.73 ± 0.07	20.49 ± 0.57	22.13 ± 0.1
SNB-75 (Glioblastoma)	19.77 ± 0.27	17.49 ± 0.15	21.9 ± 0.2	19.77 ± 0.17
SF-295 (Glioblastoma)	20.62 ± 0.34	17.01 ± 0.43	21.48 ± 0.28	21.61 ± 0.13
MCF-7 (breast adenocarcinoma)	18.49 ± 0.28	17.1 ± 0.33	20.69 ± 0.13	21.39 ± 0.24
UACC-62 (melanoma)	18.66 ± 0.8	16.31 ± 0.5	21.22 ± 0.25	20.58 ± 0.3
COR-L23P (lung carcinoma)	18.14 ± 0.34	18.12 ± 0.24	20.54 ± 0.21	21.03 ± 0.17

^a^The LightCycler RNA Master SYBR Green Kit and LightCycler 480 were used in these RT-PCR studies. The average crossing point values are given for the different cell lines ± SD (n = 4 for all samples).

^b^F-test comparing all values for PMCA4 and ATP1A1 indicates that there is a statistically significant difference (p = 0.041). No statistically significant difference is seen between PMCA4 and GAPDH (p = 0.093) or PMCA4 and HPRT1 (p = 0.074)

**Table 2:** Evaluation of ABCC1 Fold Change Using Different Housekeeping Genes^a^

**Gene Used for Relative Quantification**	**Fold Change of ABCC1 in COR-L23R cells Compared to parental COR-L23P cells**
PMCA4	90.7 ± 7.1
GAPDH	407.8 ± 98.9
HPRT1	152.3 ± 58.9

^a^The delta-delta Ct method was used with the various reference genes to calculate the fold change in ABCC1 for the COR-L23R cells compared to the parental COR-L23P cells.

**Table 3:** List of Primers used for RT-PCR

**GENE**	**Position of primer**	**Forward oligo sequence**	**Reverse oligo sequence**
ABCA2	238–684	CATCCCCCTGGTGCTGTTCTT	GCTTGGGCCGTGCTATTGG
ABCA3	437–939	GCCCTCTTTACACTCAGTTTTCA	GACGAGCAGTTGTCGTACCTAAT
ABCA4	3380–3555	TCTGGGATCTGCTCCTGAAGTATCG	GGTTAAGTACAAGCCTGTGCCAAAG
ABCB1	834–1086	GCCTGGCAGCTGGAAGACAAATAC	ATGGCCAAAATCACAAGGGTTAGC
ABCB11	2204–2371	CTTCCATCCGGCAACGCT	CACTGAATTTCAGAATCCTCCTAACTGGG
ABCC1	1119–1670	AGTGGAACCCCTCTCTGTTTAAG	CCTGATACGTCTTGGTCTTCATC
ABCC2	872–1027	AATCAGAGTCAAAGCCAAGATGCC	TAGCTTCAGTAGGAATGATTTCAGGAGCAC
ABCC3	2572–2725	TCCTTTGCCAACTTTCTCTGCAACTAT	CTGGATCATTGTCTGTCAGATCCGT
ABCC4	3880–4124	TGATGAGCCGTATGTTTTGC	CTTCGGAACGGACTTGACAT
ABCC5	3692–3864	AGAGGTGACCTTTGAGAACGCA	CTCCAGATAACTCCACCAGACGG
ABCC11	3025–3560	CCACGGCCCTGCACAACAAG	GGAATTGCCAAAAGCCACGAACA
ABCG2	266–646	CCGCGACAGTTTCCAATGACCT	GCCGAAGAGCTGCTGAGAACTGTA
ATP1A1	2237–2399	TGCGTAGTACACGGCAGTGATCTAA	CACCAGTCACAGCCACGA
GAPDH	181–587	CCCTTCATTGACCTCAACTACAT	ACGATACCAAAGTTGTCATGGAT
HPRT1	496–589	TGACACTGGCAAAACAATGCA	GGTCCTTTTCACCAGCAAGCT
PMCA4	2309–2465	ATCTGCATAGCTTACCGGGACT	TGCCAGCTTGTTTGCATTTGGCAATA
